# Comparison of the Clinical Effectiveness of Correcting Different Types of Astigmatism with Small Incision Lenticule Extraction †

**DOI:** 10.3390/jcm12216941

**Published:** 2023-11-06

**Authors:** Estera Igras, Barbara Czarnota-Nowakowska, Rónán O’Caoimh

**Affiliations:** 1 Estera Igras, Optegra Eye Health Care Laser Clinic, Mickiewicza Street 140, 71-153 Szczecin, Poland; 2 Optegra Eye Health Care Laser Clinic, 61-101 Poznan, Poland; b.czarnota@optegra.com.pl; 3 Department of Geriatric Medicine, Mercy University Hospital, Grenville Place, T12 WE28 Cork, Ireland; rocaoimh@hotmail.com; 4 Clinical Research Facility Cork, University College Cork, Mercy University Hospital, T12 WE28 Cork, Ireland

**Keywords:** myopia, astigmatism, refractive surgery, Small Incision Lenticule Extraction (SMILE), long-term effectiveness

## Abstract

Few studies have reported the differential outcomes of Small Incision Lenticule Extraction (SMILE) on myopic astigmatism. Given this, we examined the effectiveness of SMILE for up to one year, comparing with-the-rule (WTR), against-the-rule (ATR), and oblique astigmatism, conducting a retrospective review of patients who underwent correction of myopic astigmatism using the 500-kHz VisuMax femtosecond laser (Carl Zeiss Meditec) at two refractive clinics in Poland between 2016–2017. Patients were aged ≥21 with stable refractive errors between −0.5 and −10.0 diopter (D) with astigmatism up to 5D. The mean age of the 209 patients (355 eyes) available was 32 years; 58.4% were female. Of these, 247 had WTR, 62 oblique, and 46 ATR astigmatism. The mean pre-operative spherical equivalent (SE) was −5.4 ± 2.57D and the cylinder −1.7 ± 1.0D. The mean SE for WTR reduced from −5.60 ± 2.37D to −0.31 ± 0.67D at 2 months and −0.38 ± 0.70D at 12 months; the mean cylinder improved from −1.90 ± 1.10D to −0.31 ± 0.39D and −0.36 ± 0.43D, respectively. Eyes with oblique astigmatism also improved from a mean SE of −5.8 ± 3.4 D to −0.82 ± 1.50D and −0.69 ± 1.15D and a cylinder of −1.4 ± 0.73D to −0.17 ± 0.33D at 2 months and −0.1 ± 0.32D at 12. For ATR, the mean SE improved from −4.0 ± 1.8D to −0.08 ± 0.22D and −0.04 ± 0.12D; and the mean cylinder from −1.25 ± 0.53 to −0.02 ± 0.09D −0.08 ± 0.21D at 2 and 12 months, respectively. There were statistically significant improvements in SE, manifest sphere and cylinder refraction, and UDVA and CDVA scores for each cylinder type at 2 months with ATR cylinders having better outcomes. Although missing data limited interpretation at one year, differences were maintained. The magnitude of error calculations suggests that WTR was more prone to under-correction, particularly for high astigmatism (>1.5D). SMILE for myopic astigmatism reliably corrects SE, irrespective of the subtype of astigmatism.

## 1. Introduction

The choice of using Small Incision Lenticule Extraction (SMILE) for the surgical correction of refractive errors has become increasingly popular since its introduction in 2011 [1]. This flap-free intra-stromal all-in-one femtosecond laser-assisted refractive surgical procedure for the correction of myopia up to −10 diopters (D) and myopic astigmatism up to 5D is safe, effective, and has reduced rates of complications including flap-associated complications, post-operative dry eye syndrome and corneal ectasia, and aberrations associated with other keratorefractive techniques [2,3,4,5].

Although multiple studies have reported refractive outcomes after SMILE [3,4,6], few have provided results in myopic astigmatism [2,7,8,9]. Available studies show that high cylinders are prone to an under-correction of between 11–16% [2,9]. To date, only two studies have examined the differential effects of SMILE on astigmatism, both type and magnitude [10,11]. Ivarsen et al. [10] published data examining the influence of against-the-rule (ATR) and with-the-rule (WTR), but not oblique astigmatism, suggesting that SMILE induced approximately 0.35D less under-correction in ATR than WTR, independent of the attempted correction [10]. This paper also found that neither the magnitude nor the axis of the back-surface curvature impacted outcomes [10]. More recently, a smaller study of 102 eyes by Pérez-Izquierdo et al. [11] examined astigmatism correction with SMILE for all three types and found that SMILE is predictable for astigmatism lower than 1.50D without applying a correction. However, when the astigmatism is higher [≥1.50D], a correction is required but only if it is WTR [11].

Given that few studies have investigated the outcomes of SMILE on astigmatism, further investigation is required to confirm the results of Ivarsen et al. [10] and Pérez-Izquierdo et al. [11] and to examine the effectiveness of SMILE in patients with astigmatism in other populations, settings, and with more prolonged follow-up. This study compares outcomes after the correction of all three types of astigmatism, ATR, oblique, and the most common cylinder WTR [12], after SMILE at 2 months (primary analysis) and up to one year in Polish refractive laser clinics.

## 2. Materials and Methods

### 2.1. Data Collection

In this retrospective observational cohort study (case series design), the charts of consecutive patients who underwent correction of myopic astigmatism using SMILE at two laser refractive centres in Poland (Optegra^®^ Eye Health Care Clinics), between January 2016 and December 2017, were reviewed. All eyes with an attempted cylinder correction of 0.75D or higher were identified from surgical logbooks. 

### 2.2. Patients

Only patients aged ≥21 years without additional ocular pathology and with stable refractive errors (ranging between −0.5D and −10.0D) with astigmatism up to 5D were eligible for surgery and included. Only those with a baseline central corneal thickness of 500 microns were included. Medical charts were reviewed and patients were divided into three groups according to their type of astigmatism: WTR, ATR, and oblique. WTR was defined as a positive cylinder between 90° ± 29° (61° and 119°), ATR from 180° to 151° or 0° to 29°, and oblique between 120° and 150° and 30° and 60°. Each group was then divided according to the amount of astigmatism based on pre-operative subjective analysis of the cylinder axis into low astigmatism with an attempted correction from 0.75 to 2.25D and high astigmatism with a cylinder of 2.50D and greater. 

Demographic data including age and sex were collected. Data from pre-operative assessments including Uncorrected Distance Visual Acuity (UDVA), manifest, and Best Corrected Distance Visual Acuity (BCDVA) were also recorded. Distance visual acuity (VA) was measured in bright light at 6 meters (reported as 20 feet) and then recorded using the Snellen chart. Ocular residual astigmatism (ORA), an important metric to describe the discrepancy between anterior corneal astigmatism and refractive astigmatism, calculated to the corneal plane was used to quantify the astigmatism from the crystalline lens, posterior corneal surface, and retina. A trained optometrist performed the clinical refraction. All patients underwent comprehensive eye examination by the eye surgeon conducting the procedure, which included anterior segment examination, assessment of the tear film, measurement of intraocular pressure with Goldmann tonometry, and dilated fundoscopy. Corneal high-resolution Scheimpflug tomography (Pentacam-Oculus HR; Oculus Optikgeräte, Wetzlar, Germany) was performed pre-operatively. All patients were fully informed of the details and possible risks of the procedure. Written informed consent to perform the surgical procedure was obtained from all patients pre-operatively. The study was conducted in accordance with the tenets of the Declaration of Helsinki. As this was a quality control study, under Polish law, no ethical approval was required. 

### 2.3. Procedures

All surgeries were performed by three experienced refractive surgeons (EI, BCzN, and another surgeon). Pre-operatively, all eyes were anesthetized with Proxymetacaini hydrochloridum and the peripheral cornea was marked in the 0°, 90°, and 180° axis using a marker pen (Devon surgical skin marker, Covidien, Mansfield, MA 02048, USA). Under the slit lamp in the patient’s upright position, SMILE was performed using a 500-kHz Visumax femtosecond laser (Carl Zeiss Meditec, Jena, Germany). Laser settings used were as follows: cut energy of 140 nJ with a spot separation of 4.5 μm, 6.4–7.0 mm optical zone with a 0.1mm transition zone, the diameter of the cap ranged from 7.6 to 7.9 mm, and the cap thickness varied from 110 μm to 135 μm. The treatment was centered on the visual axis and the patient was instructed to look at the green flashing fixating light. Once the proper centration was achieved, the suction was applied. If any cyclotorsion (incyclotorsion or excyclotorsion) was present on the screen, a gentle rotation of the contact lens was performed to manually compensate cyclotorsion and align horizontal marks of the eye to the 0° and 180° axis of the reticule. Once all of the corneal marks were aligned, laser treatment was started to create an intra-stromal lenticule. A blunt spatula was used (Geuder G33954, Heidelberg, Germany) to break tissue bridges after the laser treatment and the lenticule was removed with a pair of forceps (Geuder G33961, Heidelberg, Germany).

Post-operative treatment included topical antibiotics (0.3% of ofloxacin) four times per day for a week, and preservative-free eye drops (Lotemax; Loteprednoli etabonas 0.5%, Bausch + Lomb, Vaughan, ON, Canada) three times a day initially and then tapered down over the next four weeks along with preservative-free artificial tears for two to three months after surgery. There were no intraoperative complications noted such as suction loss, black spots, or incomplete lenticule extraction. Post-operative follow-up examinations were scheduled for day one, one week, and two months. Surgeons who performed the surgery conducted all examinations. All follow-up visits included assessment of UDVA, manifest refraction, BCDVA, and tomography. All complications or adverse events were recorded.

### 2.4. Statistical Analysis

Data were analysed using SPSS for Mac (software version 24.0; SPSS, Inc., Chicago, IL, USA). The Shapiro–Wilk test was used to test normality. The paired Student’s *t*-test was used to compare normal data and the Wilcoxon Rank Sum Test for non-normal data; distribution frequencies were compared using Chi-Square Tests. One-way analysis-of-variance (ANOVA) was used to compare two or more independent variables. Distance VA was converted to the decimal equivalent. A modified version of the Journal of Refractive Surgery Standard for Reporting Astigmatism Outcomes of Refractive Surgery was used to report refractive outcomes [4]. Here, we used the Alpins method for astigmatism analysis [13] using the ASSORT analysis programme (individual and aggregate data analyses were performed using simple, polar, and vector analysis of astigmatism and analysis of spherical change). Vector graphs were generated using the AstigMATIC Programme [14].

## 3. Results

### 3.1. Baseline Characteristics

In all, 355 eyes from 209 patients with astigmatism of 0.75D or more were identified and included. Most, 185/355 (52%), were right eyes. Of these, 122 were female (58.4%) and 87 were male (41.6%). The median age of the sample was 32 years (interquartile range ± 5, overall range from 19 to 57 years) with a mean age of 32.9 and standard deviation (SD) of 8.1 years. At the time of the data collection, follow-up data were available for 250 eyes at 2 months and 131 at 12 months. Pre-operative (baseline), one week and two, eight and twelve-month post-operative parameters including spherical equivalent (SE), manifest sphere and cylinder, UDVA, and BCDVA are presented in Table 1. The mean pre-operative SE was −5.4D with an SD of ± 2.57D and a mean cylinder of −1.7 D (SD ± 1.0D); the mean pre-operative UDVA was 0.09 (±0.09) with a BCDVA of 0.91 (±0.15).

Eyes were categorized into three groups according to the type of astigmatism: WTR 90° ± 29° (61° and 119°), ATR from 180° to 151° or 0° to 29°, and oblique between 120° and 150° and 30° and 60°. Of those included, 247 (70%) eyes had WTR astigmatism, 62 (17.5%) eyes were oblique, and 46 (13%) eyes had ATR astigmatism. Pre-operative parameters by cylinder classification are presented in Table 2. There were statistically significant pre-operative differences in SE (*p* < 0.001), manifest sphere (*p* = 0.004), manifest cylinder refraction (*p* < 0.001), and BCDVA (*p* = 0.035) scores between all three groups but no difference in the mean UDVA scores (*p* = 0.96). The WTR group had a greater degree of pre-operative astigmatism (−1.9), compared to the oblique (−1.4) and ATR groups (−1.25).

### 3.2. Efficacy and Safety

**Figure 1:**
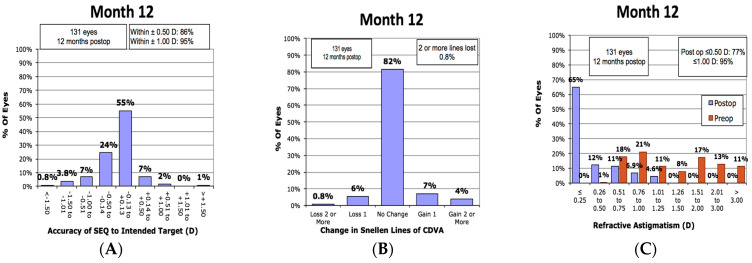

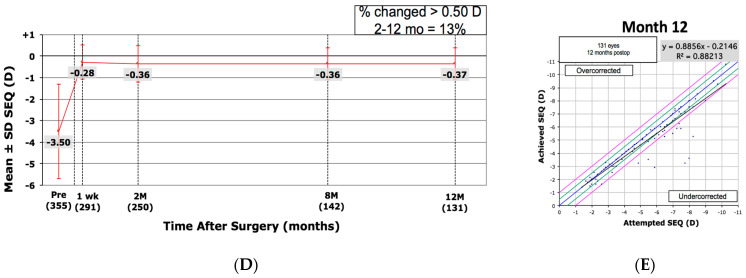
(**A**) Histogram of Spherical Equivalent Refraction Accuracy at 12 months, (**B**) Histogram comparing pre- and 12-month post-operative Best Corrected Distance Visual Acuity (BCDVA), (**C**) Histogram of the amplitude of astigmatism at 12 months, (**D**) Spherical Equivalent Refraction Stability, (**E**) linear regression for the prediction of surgically induced astigmatism vector by means of the target induced astigmatism vector at 12 months.

Target refraction was plano for the majority of eyes, 322/355 (91%); the target for the remainder ranged from −0.5D to −6.5D. The mean post-operative SE for the total sample at 2 months and 12 months was −0.36 ± 0.85D and −0.38 ± 0.75D, respectively; the mean cylinder scores were −0.18 ± 0.35D and −0.27 ± 0.40D, respectively. The mean UDVA for those included, irrespective of cylinder, was 0.87 ± 0.35D at month 2 and 0.86 ± 0.19D by month 12. At all available time points, the majority of the eyes achieved a post-operative refraction that was within 0.5D of the intended target; at 12 months 86% of eyes were within 0.50D and 95% were within 1.00D (Figure 1A). 

There was no change in BCDVA at 12 months for most patients (82% of eyes), while only 0.8% lost two or more lines of BCVA (Figure 1B). Examining the amplitude of astigmatism achieved at 12 months post-operatively, 77% of eyes were within 0.50D or less and 95% achieved 1.00D or less (Figure 1C). The mean manifest SE improved from −3.50D pre-operatively to −0.28D at one week and remained stable over 12 months follow-up (Figure 1D). Target-induced astigmatism (TIA) accounted for 86% of the surgically induced astigmatism (SIA) variation at 2 months, rising to 88% at 12 months (Figure 1E). A linear under-correction was found with its increment, slope 0.89 at 12 months. 

Post-operative parameters for all three types of cylinders over time, where available, compared with baseline, are presented in Table 3. Eyes with oblique astigmatism improved from a pre-operative mean SE of −5.8 SD ± 3.4D to −0.69 ± 1.15D with the cylinder reducing from −1.4D ± 0.73 to −0.1 ± 0.32D. For ATR, the improvement in the manifest cylinder refraction from −1.25 ± 0.53 to −0.08 ± 0.21D was greater than for WTR (−1.90 ± 1.10D to −0.36 ± 0.43D) and oblique astigmatism (−1.40 ± 0.73D to −0.10 ± 0.32D). 

Comparing refractive outcomes by cylinder type, there were statistically significant differences in SE (*p* < 0.001), manifest sphere (*p* = 0.007), manifest cylinder (*p* < 0.001), UDVA (*p* < 0.001), and BCDVA (*p* < 0.001) scores at 2 months with better outcomes for patients with ATR astigmatism. These differences remained significant at 12 months for SE (*p* = 0.02), manifest sphere (*p* = 0.017), manifest cylinder refraction (*p* = 0.002), and UDVA (*p* = 0.006) but not BCDVA (*p* = 0.08) scores. The mean pre-operative ORA for all eyes was low (0.76 ± 0.39D) and there were no statistically significant differences between the three subtypes of astigmatism (*p* = 0.279). 

**Figure 2:**
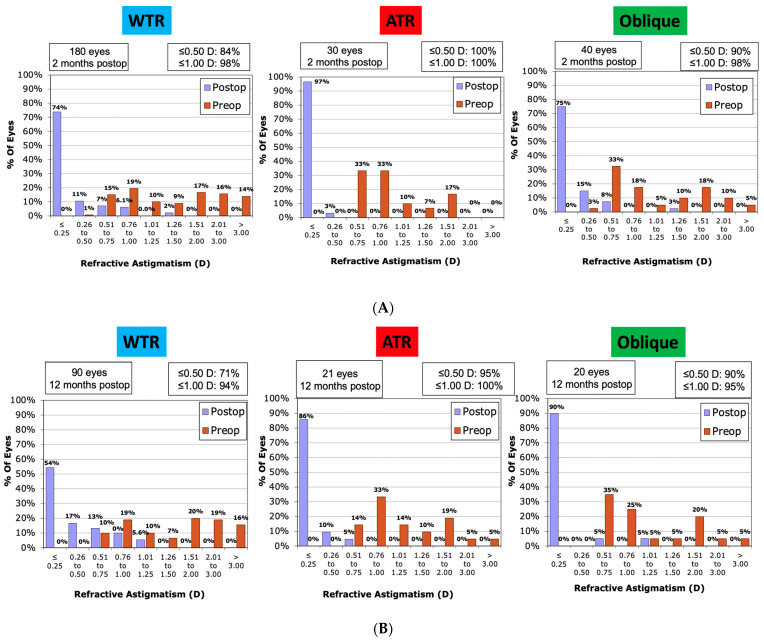
Refractive astigmatism comparing with the rule (WTR), against the rule (ATR), and oblique astigmatism; comparison at (**A**) 2 months and (**B**) 12 months.

**Figure 3:**
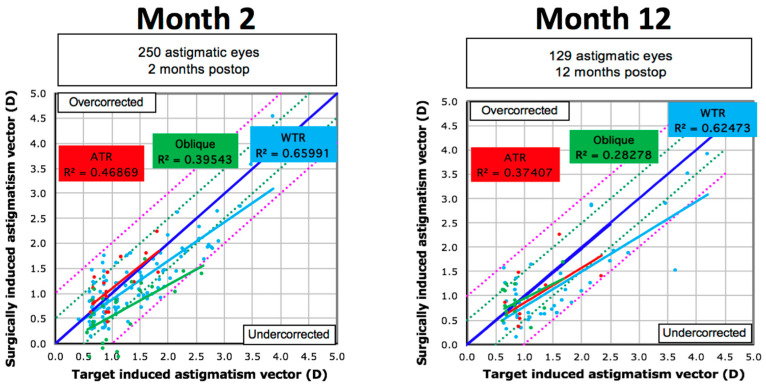
Linear regression showing the prediction of surgically induced astigmatism vector in diopter (D) by means of the target induced astigmatism vector at 2 and 12 months comparing with the rule (WTR), against the rule (ATR), and oblique astigmatism (note missing data for 2 patients at 12 months).

**Figure 4:**
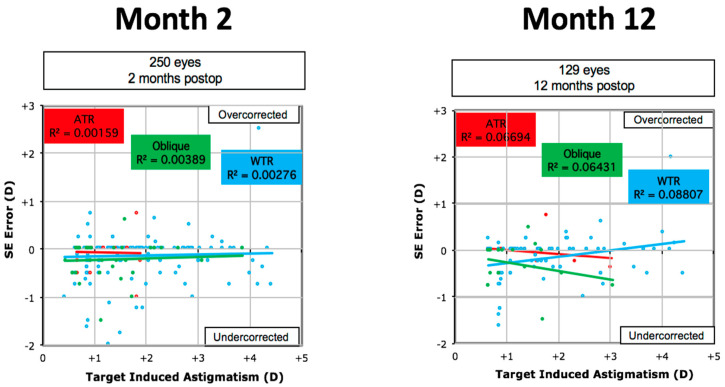
Linear regression showing the prediction of Spherical Equivalent (SE) error in diopter (D) by means of the target-induced astigmatism vector at 2 and 12 months compared with the rule (WTR), against the rule (ATR) and oblique astigmatism (note missing data for 2 patients at 12 months).

Comparing the three different types of astigmatism (WTR, ATR, and Oblique), showed that optimal correction of astigmatism was achieved for the ATR group. Of these, 100% of eyes were within ≤0.50D of target astigmatism by 2 months, while 95% remained within ≤0.50D of the target, where follow-up was available, at 12 months. Fewer (90%) of those with oblique astigmatism remained within ≤0.50D of the target at 2 and 12 months. This compared to 84% at 2 months and only 71% of the WTR astigmatism group, albeit 94% were within 1.00D at 12 months. These results are presented in Figure 2. For the WTR type, the TIA accounted for 62% of the SIA variation at 12 months versus only 37% for the ATR and 28% of the oblique groups. A small to moderate correlation was seen between TIA and SIA for all groups, largely unchanged between 2 and 12 months, though it was stronger for the WTR group (Figure 3). The Mean post-operative error in SE refraction by cylinder type is presented in Figure 4. Overall predictability was high with the mean post-op SE refraction neither correlating with the magnitude nor the axis of the intended cylinder correction. 

**Figure 5:**
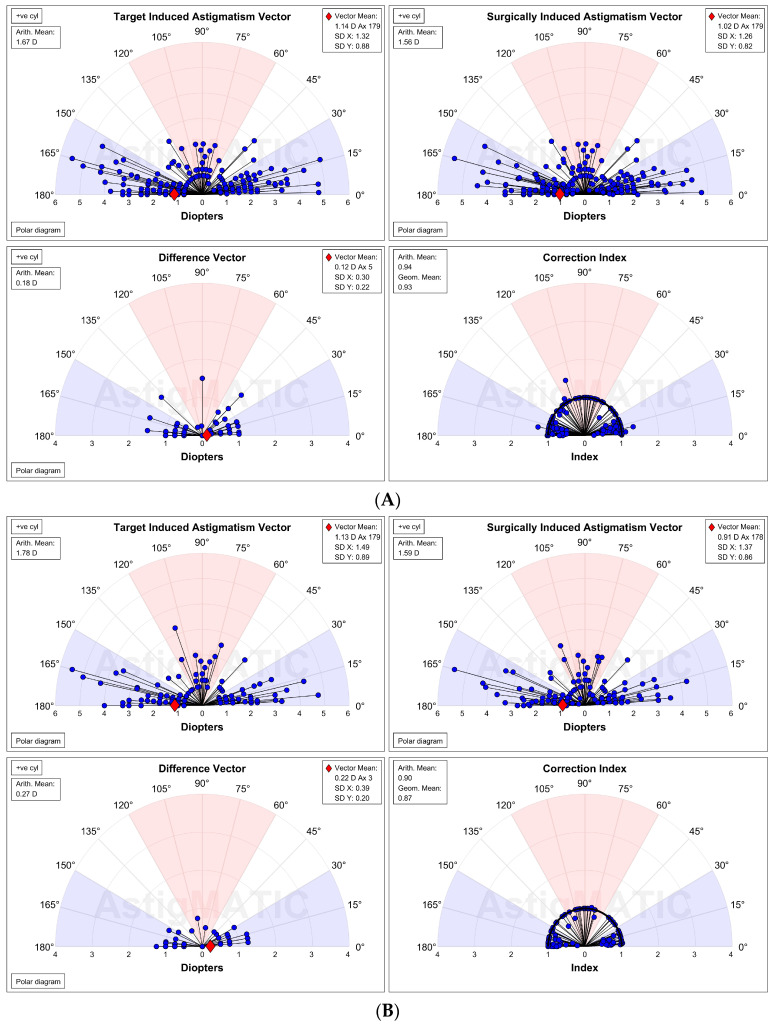
Vector graphs comparing outcomes at (**A**) 2 months and (**B**) 12 months.

The ME at 2 and 12 months for WTR was low for WTR astigmatism, irrespective of stratification by magnitude and axis of the preoperative cylinder (close to zero for those with lower manifest cylinder scores at baseline). Mean postoperative ORA remained low (<1.0 D) for all subtypes across follow-up. Vector analyses at 2 and 12 months are compared in Table 4 and Figure 5. 

Table S1, showing outcomes by low and high astigmatism, for the WTR, ATR, and oblique groups is presented in the appendix. Patients with ATR compared with WTR and oblique had better cylinder correction. Examining the magnitude of astigmatism, patients in the ATR group with higher (>2.50D) astigmatism had better results versus those with WTR astigmatism. At 8 months, post-operative SE was similar in low and high astigmatism but there is some under-correction in high WTR and low oblique groups. At 12 months, the mean post-operative cylinder in those with high astigmatism was −0.06 ± 0.18D in ATR versus −0.43 ± 0.50D in the WTR group. A similar trend was seen in low cylinder correction, although cylinder correction was stable over 12 months of follow-up in all types of astigmatism (ATR, WTR, oblique). As the correction of sphere and cylinder was better in the ATR group, the best UDVA was achieved in this group. At 12 months, post-operative UDVA in the ATR group was 0.98 ± 0.07D in those with low astigmatism and 0.85 ± 0.07D for those with high astigmatism compared with 0.81 ± 0.22D and 0.69 ± 0.30D, respectively for WTR. Few cases of oblique astigmatism with high astigmatism were available, limiting comparisons.

## 4. Discussion

This paper presents our experience of myopic astigmatism correction with SMILE in a large sample of patients (355 eyes) and in different types of astigmatism among those attending two laser refractive clinics in Poland. The results showed that SMILE provides safe and reliable correction of refractive error with mean SE and cylinder values improving for all three groups at 2 months (primary analysis). The results also suggest that SMILE likely provides long-term, at least up to one year, reliable correction of SE, irrespective of the subtype of astigmatism examined. This study adds to the existing, as yet limited literature, reaffirming data on astigmatism correction with SMILE [2,8,9]. It also adds data on outcomes relating to specific subtypes of astigmatism. Few studies have compared outcomes in different types of myopic astigmatism, particularly in such a large sample. 

In a sample of Danish patients (n = 829) in 2018, researchers showed that approximately one-quarter of the variation in outcomes with SMILE for myopic astigmatism could be accounted for by the attempted cylinder correction and the type of astigmatism (ATR versus WTR), albeit they did not provide data for oblique astigmatism [10]. However, in a smaller sample of Spanish patients in 2019 (n = 102 eyes), while higher under-correction was found for WTR astigmatism, the type of astigmatism was not associated with outcomes [11]. In our study outcomes were better for patients with ATR than the WTR or oblique astigmatism. However, these eyes had statistically significantly better pre-operative mean SE and cylinder, which may explain their scores at two-month and one-year follow-up compared to the other types of astigmatism. The results nevertheless support recent data showing that the axis and magnitude of the pre-operative cylinder influence refractive outcomes with SMILE. A recent study of 29 patients in the United States (US) showed that there is potential for under-correction in WTR with higher pre-operative cylinders and for overcorrection ATR with lower cylinders [10,15]. 

Vector analysis in our study found that the magnitude of error was significantly different for all three groups at both 2 and 12 months. In particular, the WTR group had more negative values, also suggesting that patients with WTR astigmatism may be more prone to under-correction than those with either ATR or oblique cylinders. This was especially true for those with high astigmatism. A small to moderate correlation was seen between TIA and SIA for all groups, although it was stronger for the WTR group i.e., the target TIA and SIA showed that WTE had greater correlation than ATR or oblique cylinders. This conflicts with the refractive outcomes (cylinder, SE, and sphere), which showed worse outcomes for WTR, which may be due to the small sample size of the latter and because we reported the keratometric SIA, which measures corneal astigmatism.

In our study, SE refraction stability was high with only 13% changing >0.5D by 12 months, compared to the study from the US, which found that 19% changed [15]. There was also a similar proportion (86%) with 0.5D of emmetropia at month 12, (84.4%) [16], but lower than that reported in a larger 2020 US Federal Food and Drug Administration pre-approval study of 307 eyes (95.3%) [16]. Overall predictability for this sample was high with the mean post-op SE refraction (i.e., error = achieved less attempted) neither correlating with the magnitude nor the axis of the intended cylinder correction. Post-operatively, only 0.8% of eyes with 12-month data available lost 2 or more lines of CDVA, which compares favorably with other studies [15]. Few significant adverse events were seen in this study, with none requiring intervention, reaffirming that SMILE for correction of myopia with astigmatism is safe, irrespective of the type. ORA is an important metric to assess the efficiency of astigmatism correction. There is evidence that eyes with low ORA (<1.0), are twice as likely to have effective treatment of astigmatism compared with eyes that have high pre-operative ORA (≥1.0) [17,18]. In this study, the pre-operative ORA for our sample was low with a mean of 0.76 ± 0.39D overall. It was <1.0D for all the subtypes of astigmatism and there were no significant differences between these (*p* = 0.279) at baseline. ORA for each subtype was <1.0D at both 2-month and 12-month follow-ups, supporting the efficiency of astigmatism correction with SMILE for patients in this study. 

Several studies have examined the differential effects of either Photorefractive Keratectomy (PRK) or Laser-assisted in situ keratomileusis (LASIK) versus SMILE on astigmatism. Studies examining the effectiveness of astigmatism correction with PRK and LASIK suggest that high pre-operative astigmatism is associated with lower predictability and higher risk for residual astigmatism after both LASIK and PRK [19,20]. Of the two, LASIK has achieved better results than PRK for high astigmatism correction [20,21,22]. Examining the degree of astigmatism, studies have shown that correction of eyes with high astigmatism (≥3.0D) with SMILE is comparable to the results found with LASIK [23,24]. Several studies have revealed that PRK induces an astigmatic shift toward WTR astigmatism. Similarly, the cylindrical correction showed a trend toward overcorrection for the cylindrical component for both PRK and LASIK procedures, with a statistically significant higher over-correction in PRK [20,25,26]. Induced astigmatism is generally less and more random in the axis in LASIK than in PRK; a general trend for induced WTR astigmatism in PRK was not seen in LASIK [27,28]. Studies also show that in comparison with PRK, post-operative astigmatism is significantly higher for SMILE when pre-operative astigmatism is low (<1.0D) and low when pre-operative astigmatism is more than 2.0D [29]. No difference has been found in SIA between those receiving SMILE and PRK [29]. In addition, few studies have examined long-term follow-up of different modalities on myopic astigmatism. In a study of 101 eyes from China, there were no differences in astigmatic correction between eyes treated with SMILE and LASIK at three years [30]. In another paper from Türkiye, 44 eyes showed that SMILE and LASIK had similar efficacy, predictability, and safety for up to five years [31]. To our knowledge, no other studies have examined the differential outcomes of SMILE, PRK, and LASIK on different subtypes of astigmatism. Research is therefore required in this area.

### Strengths and Limitations

The strengths of this study include its large sample size of “real-life” patients, with different types of myopic astigmatism followed for up to 12 months after SMILE in two centres in Poland, where relatively few studies have examined outcomes of SMILE. While other studies have presented outcome data, few have followed patients with astigmatism, and none, to our knowledge, with three types for up to one-year follow-up. The study has a number of limitations. There was a high degree of loss to follow-up as not all patients had yet completed 12 months of follow-up or declared they would not be attending for prolonged follow-up, which may be a source of bias including a bias towards a successful outcome. This is a common issue in cross-sectional studies and may also reflect high levels of satisfaction and, given the location of these clinics near Western European countries, a degree of “healthcare tourism”, rather than poor outcomes. Attempts to update and expand the analysis were delayed due to the COVID-19 pandemic and staff have now moved on, limiting the ability to add to the analysis. There is a wide range of targets of refraction (−0.50 to −6.50D) in this study, which may have introduced bias. This was because certain patients could only have a partial reduction of their myopia and for this reason, the target in these cases ranged widely. In all cases, the entire amount of astigmatism was treated. Here we selected 2.50D of astigmatism to categorize the low and high astigmatism groups, possibly resulting in bias. As this study was a retrospective, observational study of existing patients who were treated in our two refractive surgery clinics, the design inherently limited our sample size, the characteristics of included patients, and selection criteria. However, there is no accepted cut-off and other studies have selected either identical [2] or similar cut points [10,32]. Hence, by convenience and based on our clinical experience and that of other researchers, we divided our sample in two (<2.50D and ≥2.50D). Further, the retrospective observational nature of this study’s design limited our ability to select alternative and more widely used follow-up times. Nevertheless, the 2, 8, and 12-month intervals reflect our clinical “real-world” clinical practice, arguably increasing the generalisability of our findings. 

Most patients (70%) had WTR astigmatism, again potentially creating bias and limiting the generalisability of findings. By examining the effectiveness of SMILE for all three groups separately, we were able to compare differential outcomes and limit the potential for this. Nevertheless, at baseline, the larger WTR group had a cylinder indicating a greater degree of pre-operative astigmatism, potentially predisposing these patients to under-correction. Indeed, there were significant differences in the pre-operative astigmatism between the different subtypes examined in this paper, which could have introduced bias. Evidence suggests that a higher amount of pre-operative cylinder is associated with a greater degree of under-correction of astigmatism post-operatively [2]. Hence, given that eyes with ATR in our study had a statistically significantly better pre-operative mean cylinder, this may explain, in part, their significantly better scores compared to the other types of astigmatism. As was the case in our observational study, the WTR type of astigmatism is the most common type of astigmatism and relates to the age of the population. The over-representation of these patients may have influenced the results for the sample as a whole but as we performed a sub-analysis by type of astigmatism, we can examine the effects for each type. We found that although higher under-correction was found for WTR (as has been demonstrated in other studies), the evidence suggests that this does not necessarily influence outcomes [11]. As the sample was likely undersized to investigate the effects of SMILE on ATR and oblique and low versus high astigmatism according to subtype, further study with a larger, multi-centre study expanded sample and for a longer duration is now planned. This said, the inclusion of those willing to undergo longer follow-ups could result in selection bias [31]. As this analysis was based on a retrospective chart review, the analysis is limited by missing data including angle of error results. Further, additional patient-reported outcome measures including satisfaction could not be examined and should be performed as part of future routine care to better understand outcomes in these patients. Finally, this study did not apply nomograms. In our clinics, SMILE was introduced in 2016 for the treatment of myopic astigmatism such that the data presented here represents the first cases treated with this procedure and hence, we didn’t use nomograms as we currently do. 

## 5. Conclusions

In summary, this study affirms the growing body of evidence showing that SMILE for myopic astigmatism is an effective procedure with long-term refractive stability in a relatively large sample of patients in two refractive laser clinics in Eastern Europe (Poland), followed for up to one year. It also adds to data suggesting that the type of astigmatism as well as the cylinder are important and should be considered in pre-operative planning to provide more consistent results in high cylinder corrections. While the sample mainly included those having WTR astigmatism, almost one-third had oblique and against-the-rule cylinders, suggesting that irrespective of type, SMILE is useful for the surgical correction of myopic astigmatism. Long-term follow-up to demonstrate safety and satisfaction of SMILE for low versus high astigmatism according to subtype over a longer period of follow-up, beyond one year, is now required. 

## Figures and Tables

**Table 1:** Pre-operative and post-operative (1 week and 2, 8, and 12 months) characteristics including manifest sphere, manifest cylinder, spherical equivalent (SE), UDVA, and BCDVA.

Parameter	Eyes (n = X)	Sphere (Mean Diopter ± SD)	Cylinder (Mean Diopter ±SD)	SE (Mean Diopter ± SD)	UDVA (Mean ± SD)	BCDVA (Mean ± SD)
Timing						
Pre-operative	355	−4.50 ± 2.60	−1.70 ± 1.00	−5.40 ± 2.57	0.09 ± 0.09	0.91 ± 0.15
Week 1	355	−0.23 ± 0.80	−0.13 ± 0.29	−0.30 ± 0.80	0.82 ± 0.23	0.89 ± 0.16
Month 2	250	−0.27 ± 0.84	−0.18 ± 0.35	−0.36 ± 0.85	0.83 ± 0.24	0.91 ± 0.15
Month 8	142	−0.23 ± 0.71	−0.27 ± 0.38	−0.36 ± 0.74	0.86 ± 0.21	0.93 ± 0.13
Month 12	131	−0.24 ± 0.75	−0.27 ± 0.40	−0.38 ± 0.75	0.81 ± 0.25	0.87 ± 0.30

BCDVA, Best Corrected Distance Visual Acuity; SD, Standard Deviation; SE, Spherical Equivalent; UDVA, Uncorrected Distance Visual Acuity.

**Table 2:** Comparison of pre-operative parameters by cylinder axis: Total (n = 355 eyes) and comparison between the rule (WTR), against the rule (ATR), and oblique astigmatism.

Characteristic	Total (n = 355)	WTR (n = 247)	ATR (n = 46)	Oblique (n = 62)	*p* Value
**Spherical Equivalent** (Mean Diopter ± SD)	−5.40 ± 2.57	−5.60 ± 2.37	−4.00 ± 1.80	−5.80 ± 3.40	<0.001
**Sphere** (Mean Diopter ± SD)	−4.50 ± 2.60	−4.57 ± 2.40	−3.45 ± 1.76	−5.06 ± 3.36	0.004
**Cylinder** (Mean Diopter ± SD)	−1.70 ± 1.00	−1.90 ± 1.10	−1.25 ± 0.53	−1.40 ± 0.73	<0.001
**UDVA** (Mean ± SD)	0.09 ± 0.09	0.09 ± 0.10	0.08 ± 0.04	0.09 ± 0.09	0.96
**BCDVA** (Mean ± SD)	0.91 ± 0.15	0.91 ± 0.16	0.96 ± 0.08	0.86 ± 0.16	0.035
**ORA** (Mean ± SD)	0.76 ± 0.39	0.53 ± 0.41	0.59 ± 0.33	0.47 ± 0.31	0.279

BCDVA, Best Corrected Distance Visual Acuity; ORA, Ocular Residual Astigmatism; SD, Standard Deviation; UDVA, Uncorrected Distance Visual Acuity.

**Table 3:** Comparison of pre-operative and post-operative efficacy stratified by with the rule (WTR), against the rule (ATR) and oblique astigmatism over time.

Parameter	Sphere (Mean Diopter ± SD)	Cylinder (Mean Diopter ± SD)	SE (Mean Diopter ± SD)	UDVA (Mean ± SD)	BCDVA (Mean ± SD)
Timing					
**WTR Astigmatism**					
Pre-operative	−4.57 ± 2.40	−1.90 ± 1.10	−5.60 ± 2.37	0.09 ± 0.10	0.91 ± 0.16
Week 1	−0.17 ± 0.68	−0.16 ± 0.33	−0.24 ± 0.69	0.82 ± 0.21	0.89 ± 0.16
Month 2	−0.20 ± 0.66	−0.21 ± 0.37	−0.31 ± 0.67	0.82 ± 0.22	0.90 ± 0.16
Month 8	−0.21 ± 0.67	−0.31 ± 0.39	−0.37 ± 0.68	0.85 ± 0.21	0.94 ± 0.11
Month 12	−0.21 ± 0.72	−0.36 ± 0.43	−0.38 ± 0.70	0.78 ± 0.25	0.83 ± 0.35
**ATR Astigmatism**					
Pre-operative	−3.45 ± 1.76	−1.25 ± 0.53	−4.00 ± 1.80	0.08 ± 0.04	0.96 ± 0.08
Week 1	−0.01 ± 0.22	−0.03 ± 0.11	−0.03 ± 0.20	0.96 ± 0.11	0.98 ± 0.07
Month 2	−0.08 ± 0.22	−0.02 ± 0.09	−0.08 ± 0.22	0.97 ± 0.11	0.99 ± 0.06
Month 8	−0.01 ± 0.14	−0.13 ± 0.25	−0.08 ± 0.15	0.95 ± 0.09	0.98 ± 0.04
Month 12	0.00 ± 0.00	−0.08 ± 0.21	−0.04 ± 0.12	0.97 ± 0.08	0.99 ± 0.07
**Oblique Astigmatism**					
Pre-operative	−5.06 ± 3.36	−1.40 ± 0.73	−5.80 ± 3.40	0.09 ± 0.09	0.86 ± 0.16
Week 1	−0.67 ± 1.20	−0.06 ± 0.17	−0.70 ± 1.21	0.73 ± 0.32	0.85 ± 0.17
Month 2	−0.73 ± 1.50	−0.17 ± 0.33	−0.82 ± 1.50	0.74 ± 0.36	0.91 ± 0.13
Month 8	−0.56 ± 1.16	−0.11 ± 0.33	−0.62 ± 1.29	0.83 ± 0.30	0.87 ± 0.25
Month 12	−0.64 ± 1.06	−0.10 ± 0.32	−0.69 ± 1.15	0.80 ± 0.25	0.93 ± 0.11

**Table 4:** Vector analysis at 2 and 12 months post-operatively stratified by the magnitude and axis of pre-operative cylinder comparing with the rule (WTR), against the rule (ATR) and oblique astigmatism.

Pre-op Cylinder	Mean ± SD				
	TIA (D)	ORA (D)	SIA (D)	ME (D)	CI (D)
**2 Months**					
**WTR**					
All (n = 141)	1.51 × 105	0.55 × 103	1.24 × 111	−0.26 ± 0.73	0.92 ± 0.57
0.75–1.5 (n = 83)	0.92 × 115	0.62 × 110	0.96 × 117	0.04 ± 0.59	1.06 ± 0.68
> 1.5 (n = 58)	2.25 × 91	0.45 × 92	1.65 × 102	−0.70 ± 0.70	0.72 ± 0.27
**ATR**					
All (n = 23)	0.99 × 93	0.52 × 112	1.24 × 91	0.25 ± 0.63	1.34 ± 0.86
0.75–1.5 (n = 20)	0.87 × 93	0.51 × 111	1.15 × 92	0.28 ± 0.66	1.39 ± 0.92
> 1.5 (n = 3)	1.76 × 95	0.56 × 122	1.80 × 85	0.04 ± 0.43	1.02 ± 0.24
**Oblique**					
All (n = 31)	1.19 × 92	0.47 × 80	1.21 × 88	0.02 ± 0.65	1.21 ± 0.89
0.75–1.5 (n = 23)	0.84 × 94	0.45 × 86	1.00 × 90	0.15 ± 0.60	1.32 ± 1.00
> 1.5 (n = 8)	2.18 × 84	0.54 × 62	1.81 × 83	−0.37 ± 0.70	0.89 ± 0.25
**12 Months**					
**WTR**					
All (n = 73)	1.79 × 91	0.54 × 94	1.23 × 86	−0.56 ± 0.81	1.24 ± 3.91
0.75–1.5 (n = 35)	0.95 × 111	0.68 × 108	0.90 × 84	−0.05 ± 0.57	1.92 ± 6.51
> 1.5 (n = 38)	2.57 × 72	0.41x 81	1.54 × 87	−1.03 ± 0.71	0.61 ± 0.26
**ATR**					
All (n = 12)	1.26 × 89	0.53 × 132	0.94 × 96	−0.33 ± 0.69	0.80 ± 0.42
0.75–1.5 (n = 9)	0.90 × 90	0.44 × 140	0.72 × 102	−0.18 ± 0.37	0.81 ± 0.41
> 1.5 (n = 3)	2.35 × 87	0.79 × 110	1.59 × 78	−0.76 ± 1.31	0.78 ± 0.54
**Oblique**					
All (n = 18)	1.16 ± 94	0.43 × 76	1.29 × 83	0.1 ± 0.69	1.18 ± 0.50
0.75–1.5 (n = 13)	0.85 × 92	0.44 × 85	0.94 × 84	0.09 ± 0.51	1.17 ± 0.53
> 1.5 (n = 5)	1.95 × 98	0.40 × 51	2.19 × 80	0.11 ± 1.10	1.19 ± 0.43

CI, Correction index; D, Dioptre; ME, Magnitude of error; ORA, Ocular residual astigmatism; SIA, surgically-induced astigmatism; TIA, Target induced astigmatism.

## Data Availability

Data may be accessed on request to the corresponding author(s).

## Supplementary Materials

The following supporting information can be downloaded at: https://www.mdpi.com/article/10.3390/jcm12216941/s1, Table S1: Outcome of all three groups: with the rule (WTR), against the rule (ATR) and oblique astigmatism, comparison of low and high astigmatism groups over time.
