# Supplementary material for: Comparison of the Clinical Effectiveness of Correcting Different Types of Astigmatism with Small Incision Lenticule Extraction
Source: J Clin Med. 2023 Nov 6;12(21):6941. doi: 10.3390/jcm12216941 (PMC10648579; doi:10.3390/jcm12216941)
Supplement: Supplementary file 1 [file jcm-12-06941-s001.zip › jcm-2678842-supplementary.pdf]

Table S1. Outcome of all three groups: with the rule (WTR), against the rule (ATR) and oblique astigmatism, comparison of low and high astigmatism groups over time.

Parameter<br>Cylinder	Sphere<br>(Mean Diopter ±SD)			Cylinder<br>(Mean Diopter ±SD)			Spherical Equivalent<br>(Mean Diopter ±SD)			UDVA<br>(Mean ±SD)			BCDVA<br>(Mean ±SD)		
**Timing**	**Low**	**High** (>2.50D)	**P=X**	**Low**	**High** (>2.50D)	**P=X**	**Low**	**High** (>2.50D)	**P=X**	**Low**	**High** (>2.50D)	**P=X**	**Low**	**High** (>2.50D)	**P=X**
**WTR Astigmatism**															
**Preoperative**	-4.7 ± 2.3	-4.2 ± 2.7	0.20	-1.4 ± 0.5	-3.5 ± 1.0	<0.00	-5.4 ± 2.3	-6.0 ± 2.7	0.14	0.08 ± 0.1	0.09 ± 0.07	0.53	0.9 ± 0.13	0.8 ± 0.19	<0.00
**Week 1**	-0.13 ± 0.61	-0.24 ± 0.85	0.39	-0.1 ± 0.28	-0.3 ± 0.41	0.001	-0.18 ± 0.62	-0.39 ± 0.85	0.09	0.87 ± 0.18	0.69 ± 0.24	<0.00	0.93 ± 0.12	0.78 ± 0.21	<0.00
**Month 2**	-0.19 ± 0.6	-0.24 ± 0.81	0.69	-0.16 ± 0.28	-0.38 ± 0.53	0.01	-0.27 ± 0.62	-0.44 ± 0.80	0.21	0.85 ± 0.20	0.75 ± 0.24	0.02	0.91 ± 0.15	0.86 ± 0.19	0.125
**Month 8**	-0.22 ± 0.66	-0.19 ± 0.68	0.89	-0.26 ± 0.35	-0.48 ± 0.47	0.035	-0.34 ± 0.68	-0.43 ± 0.72	0.58	0.86 ± 0.19	0.81 ± 0.25	0.41	0.95 ± 0.10	0.90 ± 0.13	0.32
**Month 12**	-0.14 ± 0.45	-0.38 ± 1.2	0.32	-0.33 ± 0.40	-0.43 ± 0.50	0.36	-0.30 ± 0.45	-0.60 ± 1.1	0.20	0.81 ± 0.22	0.69 ± 0.30	0.10	0.84 ± 0.39	0.82 ± 0.22	0.76
**ATR Astigmatism**															
**Preoperative**	-3.5 ± 1.8	-1.6 ± 0.18	<0.00	-1.15 ± 0.39	-2.88 ± 0.53	<0.00	-4.1 ± 1.86	-3.1 ± 0.18	0.01	0.09 ± 0.05	-0.05 ± 0.04	0.004	-0.97 ± 0.08	0.85 ± 0.07	0.25
**Week 1**	-0.01 ± 0.22	NA	NA	-0.03 ± 0.11	NA	NA	-0.03 ± 0.2	NA	NA	0.96 ± 0.11	NA	NA	0.98 ± 0.07	NA	NA
**Month 2**	-0.08 ± 0.22	NA	NA	-0.02 ± 0.09	NA	NA	-0.08 ± 0.22	NA	NA	0.97 ± 0.11	NA	NA	0.99 ± 0.06	NA	NA
**Month 8**	-0.01 ± 0.14	NA	NA	-0.13 ± 0.25	NA	NA	-0.08 ± 0.15	NA	NA	0.95 ± 0.09	NA	NA	0.98 ± 0.04	NA	NA
**Month 12**	0.0 ± 0.0	-0.0 ± 0.0	NA	-0.03 ± 0.11	-0.06 ± 0.18	0.12	-0.01 ± 0.06	-0.03 ± 0.09	0.12	0.98 ± 0.07	0.85 ± 0.07	0.21	0.98 ± 0.07	1.0 ± 0.0	NA
**Oblique Astigmatism**															
**Preoperative**	-5.0 ± 3.6	-5.5 ± 1.5	0.47	-1.2 ± 0.41	-2.7 ± 1.13	<0.00	-5.7 ± 3.6	-6.8 ± 1.6	0.13	-0.09 ± 0.08	-0.04 ± 0.02	0.004	0.89 ± 0.16	0.88 ± 0.16	0.85
**Week 1**	-0.73 ± 1.3	-0.25 ± 0.55	0.09	-0.05 ± 0.16	-0.13 ± 0.23	0.40	-0.76 ± 1.3	-0.31 ± 0.56	0.16	0.71 ± 0.34	0.79 ± 0.22	0.44	0.85 ± 0.18	0.90 ± 0.14	0.37
**Month 2**	-0.87 ± 1.63	-0.19 ± 0.44	0.045	-0.20 ± 0.36	-0.06 ± 0.18	0.15	-0.97 ± 1.63	-0.22 ± 0.53	0.04	0.72 ± 0.37	0.80 ± 0.27	0.268	0.91 ± 0.14	0.90 ± 0.13	0.88
**Month 8**	-0.62 ± 1.3	-0.33 ± 0.58	0.60	-0.14 ± 0.32	-0.12 ± 0.30	0.22	-0.68 ± 1.42	-0.33 ± 0.58	0.52	0.82 ± 0.33	0.83 ± 0.32	0.88	0.86 ± 0.28	0.89 ± 0.22	0.86
**Month 12**	-0.57 ± 1.1	-1.24 ± 1.06	0.52	-0.11 ± 0.33	0.0 ± 0.0	NA	-0.62 ± 1.2	-1.25 ± 1.1	0.55	0.81 ± 0.30	0.84 ± 0.32	0.92	0.94 ± 0.11	0.88 ± 0.14	0.38

BCDVA = Best Correct Distance Visual Acuity
NA = Not Available (numbers too small to analyze)
SD = Standard Deviation
UDVA = Uncorrected Distance Visual Acuity
